# 3D Culture Platform for Enabling Large-Scale Imaging and Control of Cell Distribution into Complex Shapes by Combining 3D Printing with a Cube Device

**DOI:** 10.3390/mi13020156

**Published:** 2022-01-21

**Authors:** Atsushi Takano, Isabel Koh, Masaya Hagiwara

**Affiliations:** 1 Cluster for Pioneering Research, RIKEN, Saitama 351-0198, Japan; atsushi.takano@riken.jp (A.T.); isabelsiewyin.koh@riken.jp (I.K.); 2 Department of Biological Science, Osaka Prefecture University, Osaka 599-8531, Japan

**Keywords:** 3D culture, 3D imaging, bioprinter, organoid, 3D control, carbohydrate glass

## Abstract

While organoid differentiation protocols have been widely developed, local control of initial cell seeding position and imaging of large-scale organoid samples with high resolution remain challenging. 3D bioprinting is an effective method to achieve control of cell positioning, but existing methods mainly rely on the use of synthetic hydrogels that could compromise the native morphogenesis of organoids. To address this problem, we developed a 3D culture platform that combines 3D printing with a cube device to enable an unrestricted range of designs to be formed in biological hydrogels. We demonstrated the formation of channels in collagen hydrogel in the cube device via a molding process using a 3D-printed water-soluble mold. The mold is first placed in uncured hydrogel solution, then easily removed by immersion in water after the gel around it has cured, thus creating a mold-shaped gap in the hydrogel. At the same time, the difficulty in obtaining high-resolution imaging on a large scale can also be solved as the cube device allows us to scan the tissue sample from multiple directions, so that the imaging quality can be enhanced without having to rely on higher-end microscopes. Using this developed technology, we demonstrated (1) mimicking vascular structure by seeding HUVEC on the inner walls of helix-shaped channels in collagen gels, and (2) multi-directional imaging of the vascular structure in the cube device. Thus, this paper describes a concerted method that simultaneously allows for the precise control of cell positioning in hydrogels for organoid morphogenesis, and the imaging of large-sized organoid samples. It is expected that the platform developed here can lead to advancements in organoid technology to generate organoids with more sophisticated structures.

## 1. Introduction

Organoids are in vitro mini organs that mimic some of the functions of in vivo tissues or organs and are highly expected to contribute to applications such as drug discovery, regenerative medicine, and as models to study developmental biology [1,2,3]. Nevertheless, one of the bottlenecks of current organoid culture methods that could be hindering further progress in the field is the lack of control of the shape of organoids. In most cases, organoids are cultured simply as a cluster of cells, either free-floating or in an extracellular matrix (ECM) hydrogel, and allowed to self-organize with only molecular cues for guidance. However, this oversimplified method of culturing organoids does not take into account the physical boundaries and the local environment surrounding the cells that contribute important cues for the formation and function of organoids, such as mechanical stress distribution in tissues and ECM composition [4,5]. Several methods have been employed to enhance the control of organoid shape, including micromanufacturing hydrogels with pre-defined shapes [6,7], laser-ablation of microchannels in hydrogels [8], and utilizing microfilaments as scaffolds for cells to attach onto [9,10]. Still, the patterns and shapes that can be achieved by these technologies are mainly limited to simple linear geometries only.

On the other hand, advances in 3D-printing technologies have greatly increased the complexity and sophistication of configurations in which cells can be formed into organoids [11,12]. Sacrificial templating, in which a sacrificial material is used to provide temporary structural support during the fabrication process, has been widely applied to create complex 3D tissues. For example, the freeform reversible embedding of suspended hydrogels (FRESH) method was used with a gelatin support material to fabricate a heart ventricle using collagen and cell-laden bio-inks [13], and water-soluble Pluronic or carbohydrate glass inks have been used as sacrificial molds to create vascular channels in tissue constructs [14,15]. However, due to the relatively large sizes of the fabricated 3D tissues or organoids, imaging resolution in the *z*-direction tends to be poor. Cryo- or paraffin-sectioning methods are commonly used to visualize deeper sections of large tissue samples, but not for obtaining z information, as it is difficult to reconstitute a whole image of the sample from the sliced sections. On the other hand, high-resolution images can be obtained with higher magnification lenses, but the focal depths of these objective lenses are only around tens to hundreds of micrometers, which is inadequate for samples that are of millimeter order in size. Inversely, low magnification lenses have larger focal depths but with compromised resolution. To overcome this issue, clearing reagents are often used to render samples transparent to enable deeper scanning of the sample with a laser, but the focal depth issue remains when a low magnification lens is used.

**Figure 1:**
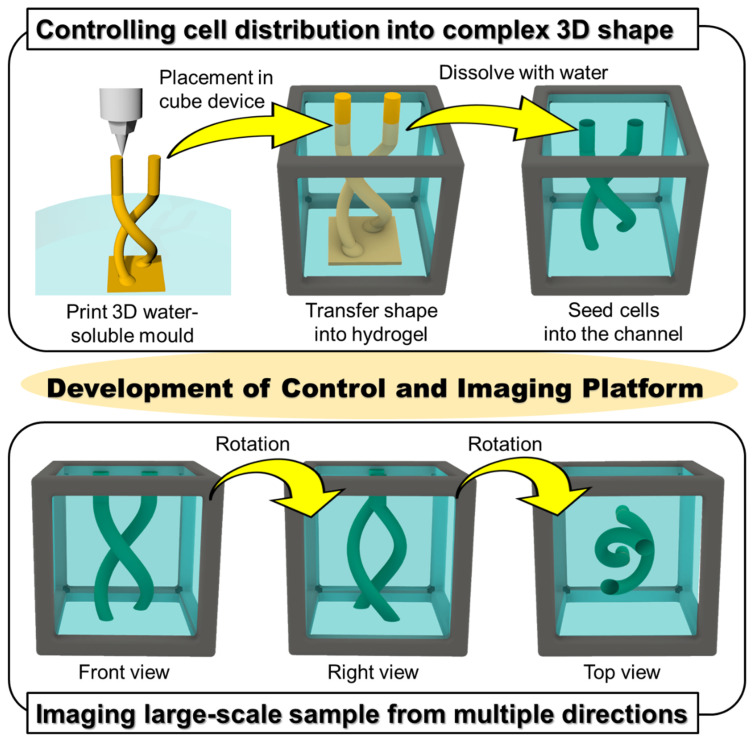
Conceptual images of 3D culture platform to enable the control of cell distribution into complex shapes and large-scale imaging. Water-soluble material (carbohydrate glass) allows us to fabricate complex-shaped molds and cells can be seeded in the channel developed by the mold in a cube device. The cube allows us to scan from multiple directions by rotating the cube so that large-scale imaging can be achieved.

We previously developed a gel cube device made of a polycarbonate frame, outer agarose gel walls, and an inner ECM hydrogel in which tissue samples are supported [16]. We showed that this cube device can be used to control initial cell seeding geometry with simple shapes such as cylinders or prisms for highly repeatable experiments [6] and to obtain high-resolution imaging on a large-scale by acquiring and merging images of the sample from all six sides of the cube [17]. In this paper, to develop a platform that simultaneously allows cell seeding control and high-resolution imaging of large-scale samples for organoid culture, we utilized 3D-printed sacrificial molds to generate more complex patterns in the hydrogel in the cube device compared with the limited simple patterns obtainable by photocurable resin molds in our previous work. We opted to use carbohydrate glass originally developed by Miller et al. [15,18], which is now commercially available, as the non-cytotoxic resin for our molds. To make complex shapes in the cube device, printed molds of the desired design were placed in the hydrogel in the cube device, and once the gel cures, the mold can be removed simply by immersing the cube in an aqueous solution, leaving only a patterned shape in the hydrogel (Figure 1). We also designed and fabricated accessories using a stereolithography 3D printer to align the position of the mold in the cube, which facilitates the precise transfer of the mold shape to the hydrogel. We first optimized the printing parameters that would enable us to accurately print the sacrificial mold structures, then compared the printed mold dimensions with that of the pattern formed in the hydrogel to validate the preservation of structural accuracy after transfer of the mold shape to the hydrogel. Finally, to demonstrate the advantages of integrating sacrificial templating with the gel cube device, we seeded endothelial cells in the formed shape, and performed multi-directional imaging to visualize the tissue construct.

## 2. Materials and Methods

### 2.1. Materials

Polydimethylsiloxane (PDMS; Silpot 184, Dow Corning, Midland, MI, USA) was used to make the PDMS sidewalls of the gel cube device and flexible silicone printing surfaces. Water-soluble carbohydrate glass (CG3357, Volumetric, Houston, TX, USA) was used for mold printing. Poly (D, L-lactide-co-glycolide) (PLGA; 26269-10, Polysciences, Warrington, PA, USA) and chloroform (08402-55, Nacalai Tesque, Kyoto, Japan) were used to prepare PLGA solution as a hydrophobic coating for the mold. The compound 2-propanol (29112-63, FUJIFILM Wako Pure Chemical, Osaka, Japapn) was used for cleaning the cube devices and to remove excess PLGA coating. An amount of 3 mg/mL collagen type I derived from porcine tendon (Cellmatrix type IA, Nitta Gelatin, Morrisville, NC, USA) was used as a hydrogel. Phosphate-buffered saline (PBS; 14190-144, Gibco, Waltham, MA, USA) was used for the dissolution of water-soluble molds, retention of hydrogel, and in cell culture. Human umbilical vein endothelial cells (HUVEC; C2519A, Lonza, Basel, Switzerland), EGM-2 medium (CC-3162, Lonza), penicillin–streptomycin (Pen-strep; 15140-122, Gibco), Trypsin-EDTA (25200-056, Gibco), and Trypsin neutralizing solution (TNS; HK-3220, Kurabo, Osaka, Japan) were used in cell culture. Alexa fluor 488 Phalloidin (A12379, Thermo Fisher Scientific, Waltham, MA, USA) was used for immunofluorescence staining. Fluorescent microbeads with 45 µm diameter (18242-2, Polysciences, Warrington, PA, USA) were used for measurements of the gel channels.

### 2.2. Preparation of Cube Device

A number of 10 mm-sized cube frames were made by machining. To make a PDMS sidewall, the frames were cleaned with 2-propanol followed by deionized water, for 10 min each with ultrasonication and then dried by blowing with compressed air. The frames were placed in an oven at 85 °C for 1 h to ensure the frames were free of moisture. The frames were placed on an uncured PDMS-coated surface of a 100 mm polystyrene dish. After the dish was degassed at −0.08 MPa for 30 min, the dish was placed in the oven at 85 °C for 1 h to cure the PDMS. The PDMS surface was trimmed with a scalpel along the frame to form the PDMS sidewall. The process was repeated three more times with the frame rotated 90° each time to cover the other three lateral surfaces with PDMS in the same manner.

### 2.3. 3D Printing of Water-Soluble Mold

Molds were designed with a 3D modelling software (Rhinoceros 3D, McNeel, Seattle, WA, USA) and exported to G-codes with a 3D printing software (Repetier-host, Hot-World, Willich, Germany). The molds were then printed on a PDMS surface with a bioprinter (Allevi 1, Allevi, Philadelphia, PA, USA) using carbohydrate glass. Configurations of the bioprinter’s dispensing unit consisted of a 5 mL steel cylinder and a φ100 μm dispenser nozzle (SHN-0.1N, Musashi Engineering, Tokyo, Japan). To print the molds, optimized printing conditions of printing speed, discharge pressure, and printing temperature were 5 mm/s, 70–80 PSI, and 150 °C, respectively. Printed molds can be stored for a few days in a dehumidifying cabinet (ND-1S, AS One, Osaka, Japan) below 30% relative humidity to prevent the water-soluble molds from dissolving.

### 2.4. Hydrophobic Coating

Before hydrophobic coating, the molds were put in a cell culture CO_2_ incubator (5% pCO_2_, 95% humidity, 37 °C) for 30 min to smoothen the surface of the mold and then the molds were stored in the dry cabinet for a few hours to remove moisture from the mold. Once dry, the mold was immersed in 50 mg/mL PLGA solution for 3 min to make a hydrophobic coating on the mold. Immediately after that, the mold was immersed in 2-propanol for 1 min to remove excess PLGA, and 2-propanol was removed from the mold by blowing with compressed air. After the hydrophobic coating, the mold was bonded, using 5 µL of 50 mg/mL PLGA solution, to a cube holder that was printed using AR-M2 resin with a stereolithography 3D printer (Agilista 3200, Keyence, Osaka, Japan), then stored in the dry cabinet for at least 3 h to allow PLGA to adhere to the mold.

### 2.5. Molding

A mold holder was also 3D-printed using AR-M2 resin, to hold the mold in place in the cube. Before transferring the mold into the hydrogel, the cube device was exposed to vacuum plasma (−10 kPa, 3 min) with a plasma treater (PIB-10, vacuum device) to hydrophilize the cube device to prevent the generation of bubbles in the corners of the cube device when hydrogel is added into the cube. The molding setup in preparation for hydrogel injection was assembled together as three components: the hydrophobic coated mold attached to the cube holder on the bottom, the cube device placed on the cube holder, and the mold holder on top of the cube. Collagen solution prepared according to manufacturer’s protocol at 4 °C was then injected into the cube device from the top side of the setup. After curing the collagen for 30 min on a hotplate at 37 °C, the setup was immersed in PBS to dissolve the water-soluble mold for at least 90 min in a CO_2_ incubator. Then, the cube holder and mold holder were removed from the cube device while still immersed in PBS bath, before being transferred to fresh PBS. The cube in PBS was placed on a see-saw shaker (NA-M101, Nissin, Tokyo, Japan) for 10 min to wash out residual mold.

### 2.6. Cell Seeding

HUVEC was cultured in EGM-2 and used at passage 4. To prepare the cell suspension of HUVEC, HUVEC around 80% confluency was washed with PBS, then treated with Trypsin-EDTA for 2.5 min at 37 °C before neutralizing the trypsin with TNS. Collected HUVEC was centrifuged at 300× *g*, 4 °C for 4 min. After aspirating the supernatant, cell density was adjusted to 30 × 10^6^ cells/mL using EGM-2. Before cell seeding, the cube device with the inlets of channels facing up was placed on a glass slide and the outer surfaces of the cube device were wiped gently with a Kimwipe wipe to remove excess moisture. An amount of 10 µL of the HUVEC suspension was injected into the inlets of the channels. After confirming that the HUVEC suspension had flowed through the channel, the cube device was rotated 90° and transferred to a 12-well plate. The cube device was incubated in a CO_2_ incubator for 30 min to allow the HUVEC to adhere to the inner wall of the channels. Then, the cube device was rotated 180° and incubated for another 30 min for the HUVEC to adhere to the opposite wall of the channel. The rotation and incubation were repeated twice in the same way. Then, the cube device, with the inlets facing up, was placed on a slide glass again and another 10 µL of fresh HUVEC suspension at 30 × 10^6^ cells/mL was injected and the rotation-incubation procedure repeated to enable the HUVEC to adhere to the two remaining surfaces of the channel, so that the entire inner wall of the channel is seeded with cells. After seeding, the cells were cultured for 5 days in 2 mL of EGM-2 in a 24-well plate on a see-saw shaker in a CO_2_ incubator. Every day, the cube device was rotated 90°, and 1 mL of EGM-2 was replaced with fresh medium.

### 2.7. Immunofluorescence Staining

Samples were washed with PBS for 5 min and fixed with 4% PFA for 20 min at room temperature. Then, the samples were washed with PBS for 5 min at room temperature before permeabilization with 0.5% Triton X-100 for 10 min at 4 °C. After rinsing with PBS three times, the samples were treated with 100 mM glycine for 15 min at room temperature and rinsed once with PBS. Blocking was performed for 45 min at room temperature with IF-buffer (10% Goat serum, 0.2% Triton X-100, 0.1% BSA, and 0.05% Tween-20 in PBS), and then stained with Alexa fluor 488 Phalloidin (1:200) for 45 min at room temperature. The samples were then washed three times with PBS for 5 min at room temperature before imaging.

### 2.8. Measurement of Water-Soluble Molds

3D-printed molds were imaged with a phase-contrast microscope (CKX41, Olympus, Waltham, MA, USA). Dimensions of the molds were measured from the acquired phase-contrast images with open-source image processing software (ImageJ, NIH, Bethesda, MD, USA).

### 2.9. Measurement of Channels in Gel

Polystyrene-latex fluorescent beads (45 µm diameter, excitation wavelength = 441.53 nm, emission wavelength = 485.56 nm) diluted in 1.5% agarose solution was injected into the channels formed in the collagen gel in the cube device and cooled for 3 min at 4 °C. *Z*-stack images of the channels were taken from multiple directions with a fluorescence microscope (BZX-700, Keyence, Osaka, Japan) and *z*-projection images were exported to ImageJ for measurement analyses.

### 2.10. Multi-Directional Imaging

*Z*-stack images of fluorescently stained HUVEC in the cube device were taken from multiple directions with the fluorescence microscope with sectioning function by rotating the cube device and taking images from each of its sides. The *z*-stack images were overlaid with an image alignment software that was developed in-house and then exported as *z*-projection images using ImageJ.

## 3. Results 

### 3.1. Experimental Design of the Control of Cell Position in Cube Device

To establish the method for designing and controlling cell position in the cube device, a molding process was developed. From the concept described above (Figure 1), it is critical to control the shape and positioning of the mold in the hydrogel in order to transfer the design to the hydrogel with high reproducibility. Hence, attention needs to be given not only to designing the desired final shape, but also to the accessory parts that contribute to the alignment and positioning of the mold in the cube device. Below, we detail the design principles and experimental procedures to control the design of cell positioning in hydrogel in the cube.

**Figure 2:**
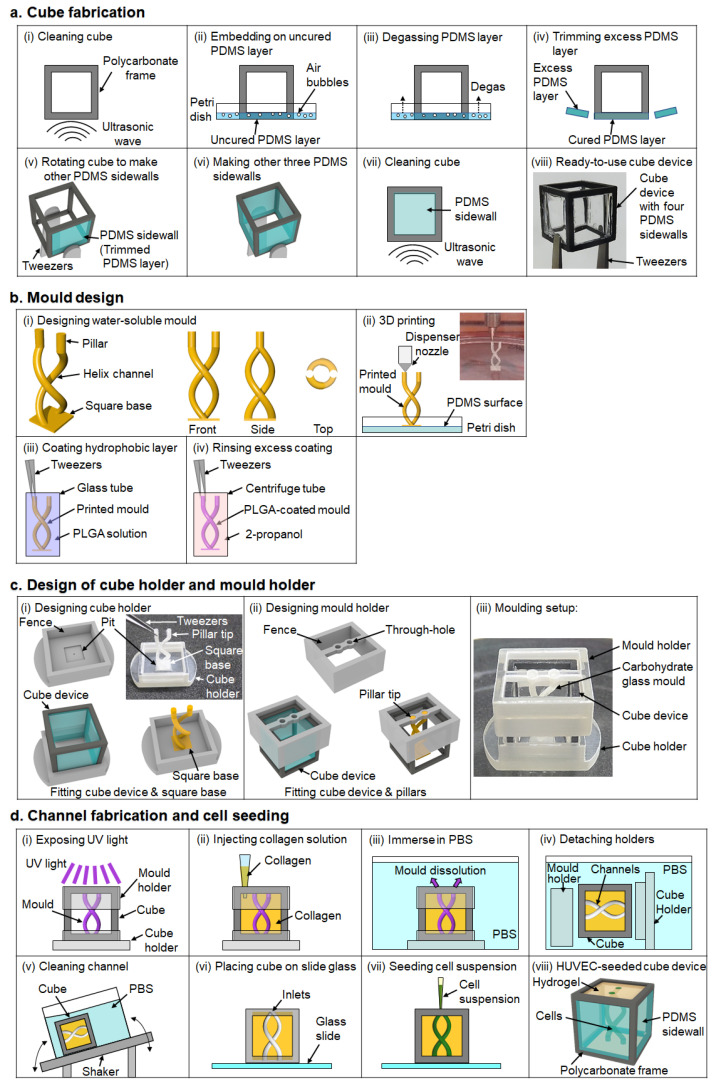
Fabrication processes to control cell distribution in a cube. (**a**) Cube device fabrication. (**b**) Fabrication process of the carbohydrate mold. (**c**) Design of the accessories to align the mold with a cube. (**d**) Process of transferring mold design into ECM to seed cells in a cube.

Figure 2a shows the process of making a cube device with PDMS sidewalls. Compared with a cube device with agarose gel sidewalls reported in our previous studies [16,17], cubes with PDMS sidewalls are more robust and better able to withstand frequent handling with tweezers, plus hydrogels are less likely to detach from the PDMS walls. Hence, the sidewall preparation protocol was modified in this study. Before fabricating the sidewalls of the cube device, the polycarbonate frame was cleaned by ultrasonication in 2-propanol and deionized water (Figure 2a(i)), then dried in an oven. Once dry, the frame was placed on an uncured layer of PDMS to cover one side of the frame with PDMS (Figure 2a(ii)). Since air bubbles contained in the PDMS layer can cause a decrease in the robustness and optical properties of the PDMS sidewall, the uncured PDMS was degassed to remove air bubbles (Figure 2a(iii)) before placing in an oven to cure the PDMS. After curing the PDMS layer, excess PDMS was trimmed from the frame to form the PDMS sidewall (Figure 2a(iv)). Then, the cube device was rotated, and the process was repeated for the remaining three lateral sidewalls (Figure 2a(v,vi)). The cube device with four PDMS sidewalls was cleaned by ultrasonication and dried again before use (Figure 2a(vii,viii)).

The principles of designing the water-soluble mold are shown in Figure 2b. The mold was designed as a helix-shaped structure comprising two curved channels with straight pillars on one end of the channels, and the other ends attached to a square base (Figure 2b(i)). The square base was required not only as a foundation of the 3D-printed mold, but also serves to create a tapered aperture leading to the inlets of the channels. On the opposite side, the pillars were designed not only for holding the mold in place by insertion into a mold holder, but also as outlets of the channels. Figure 2b(ii) shows the 3D printing of carbohydrate glass. Carbohydrate glass is a sticky material so the printing surface on the bottom of a Petri dish must be coated with PDMS for easy removal after printing. Once the molds are printed, they are exposed to moisture by placing in a CO_2_ incubator to smoothen the surface of the mold. Thereafter, a hydrophobic coating is also required because carbohydrate glass dissolves quickly in hydrogel solutions. The hydrophobic coating solution used was PLGA dissolved in chloroform, which was dispensed into a glass tube. The mold was picked up by the pillar tip with a pair of tweezers and dipped in the PLGA solution (Figure 2b(iii)). As the pillar tip is not immersed in hydrogel during molding, the parts that are not coated with hydrophobic coating due to being held by the tweezers will not be affected during the molding process. After dipping in PLGA, the mold was immediately immersed in 2-propanol in a centrifuge tube to remove excess PLGA, then blown dry with compressed air (Figure 2b(iv)).

The principles of designing the cube and mold holders that facilitate the alignment of the mold in the cube device are shown in Figure 2c. The cube holder was designed with a fence to ensure the cube is held in place, and a pit that fits the square base of the mold to fix the position of the printed mold (Figure 2c(i)). The mold is glued to the cube holder by dripping PLGA solution into the pit, then placing the square base of the mold in the pit. The cube holder not only reproducibly controls the spatial positioning of the mold, but also prevents leaks from the bottom of the cube device when the hydrogel solution is injected into the cube device. The mold holder was similarly designed with a fence part to fit on the cube, and through-holes to hold the pillars of the mold so that the mold does not tilt out of place before the hydrogel is fully cured (Figure 2c(ii)). The entire molding setup, with the cube holder, cube device, carbohydrate glass mold, and mold holder is shown in Figure 2c(iii).

Figure 2d shows the process of forming the helix channels inside the hydrogel in the cube device, and the seeding of endothelial cells in the channels. The molding setup was first UV sterilized (Figure 2d(i)). Then, hydrogel gel solution was gently injected into the cube device through the aperture of the mold holder (Figure 2d(ii)). After curing the hydrogel solution, the molding setup was immersed in a PBS bath to dissolve the mold (Figure 2d(iii)), and later the cube device was removed from the molding setup after rotating the setup by 90° in the PBS bath (Figure 2d(iv)). Although carbohydrate glass can be dissolved quickly, some layers of PLGA coating still remained in the channel. Thus, the cube device was cleaned by placing the cube in PBS on a shaker, with the PDMS sidewall facing downwards and the inlets and outlets aligned with the direction of PBS flow to wash out residual mold and PLGA coating (Figure 2d(v)). For cell seeding, the cube device was placed on a glass slide with the inlets facing upwards, excess moisture removed with a Kimwipe wipe, and HUVEC suspension was injected into the inlets of the channel (Figure 2d(vi,vii)). By these processes, the designed channel shape can be formed accurately and reproducibly in the cube device, and cells can be positioned in the formed channels in the cube (Figure 2d(viii)).

### 3.2. Printing Accuracy of Carbohydrate Glass Mold

**Figure 3:**
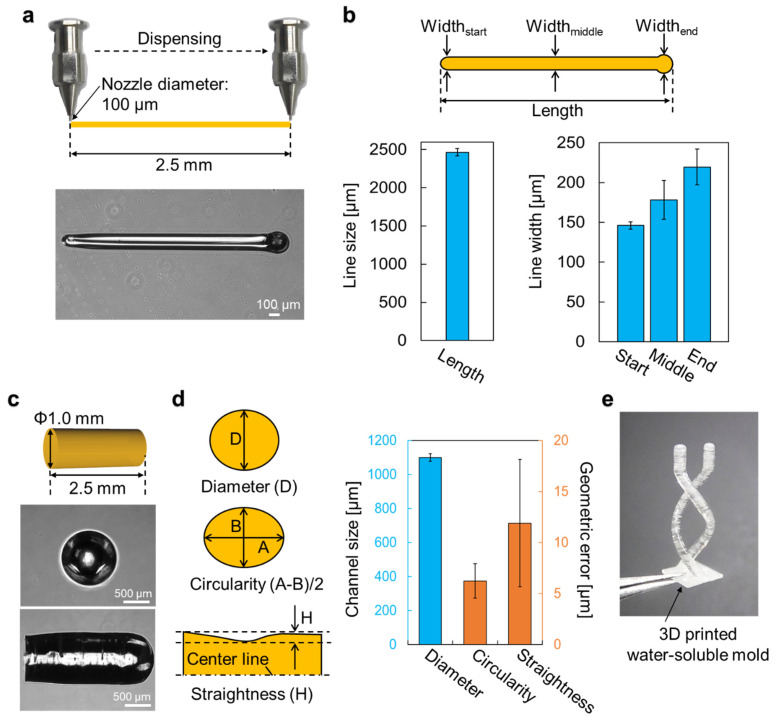
Measurement results of carbohydrate mold accuracy. (**a**) Measurement of the dispensed straight line of carbohydrate glass on a dish. (**b**) Measurement results of the straight line on a dish (*n* = 5). (**c**) Measurement of the pillar made by carbohydrate glass. (**d**) Measurement results of the shape of the pillar (*n* = 5). (**e**) Helix-shaped printed mold. Error bars denote standard deviation.

The printing accuracy of the carbohydrate glass mold is a major factor to take into account to control multi-cellular formation with this fabrication process. In general, the accuracy of printing highly depends on the printing material, ejection pressure, stage velocity, and the pitch in the *z* direction. Additionally, carbohydrate glass is a viscous liquid above 110 °C, but it hardens quickly when cooled down. Therefore, the balance between the temperature of the cartridge holding the printing material, nozzle size, and ejection speed from the cartridge affects printing accuracy as well. Hence, before printing complex shapes such as helix-shaped channels, we needed to optimize these parameters for carbohydrate glass printing and to confirm the accuracy of the printed mold. We first printed a simple straight line with a length of 2.5 mm on a Petri dish using a 100 μm diameter dispensing nozzle to confirm the dispensing accuracy (Figure 3a). The measured length of the printed line was about 2465.7 μm, and the widths at the start, middle, and end of the line were 146.1 ± 4.7 μm, 178.4 ± 24.4 μm, and 219.5 ± 22.4 μm (mean ± standard deviation, *n* = 5), respectively (Figure 3b). Although lower ejection pressures can generate line widths that are closer to that of the diameter of the nozzle, ejection becomes unstable at pressures lower than 70 psi. Additionally, the variation is quite small, with standard deviation less than 25 μm. The variations at the start and end points of the line were due to the initial increase in ejection pressure at the start of printing, and an excess droplet at the end of printing, respectively. Nevertheless, the data shows consistent results with standard deviations of 4.7 μm, 24.4 μm, and 22.4 μm at start, middle, and end points, respectively.

We next printed a pillar-shaped mold with a diameter of 1.0 mm and height of 2.5 mm (Figure 3c) to measure the accuracy in printing tall structures, as the effects of gravity may deteriorate printing accuracy during the lamination of carbohydrate glass to obtain height. The diameters of the pillar structures were on average 1100.1 μm (Figure 3d), which is 100 μm larger than the designed size, but the standard deviation is only 22.4 μm. Geometric dimensions were measured as circularity ((A−B)/2) and straightness (r_max_−r_min_) of the pillar, which were 6.23 ± 1.7 μm and 11.9 ± 6.2 μm (mean ± standard deviation, *n* = 5), respectively (Figure 3d). As the variations measured were all in micrometer order, which is only around the size of 2–4 cells, we decided that the order of accuracy of the printed carbohydrate glass molds was sufficient for our purposes to control multi-cellular organization which is in the millimeter order. To demonstrate that complex shapes can also be patterned in hydrogels by using this method, we designed and printed a helix-shaped mold to be used in the experiments that follow (Figure 3e).

### 3.3. Accuracy of Patterned Shape after Transfer to Hydrogel

**Figure 4:**
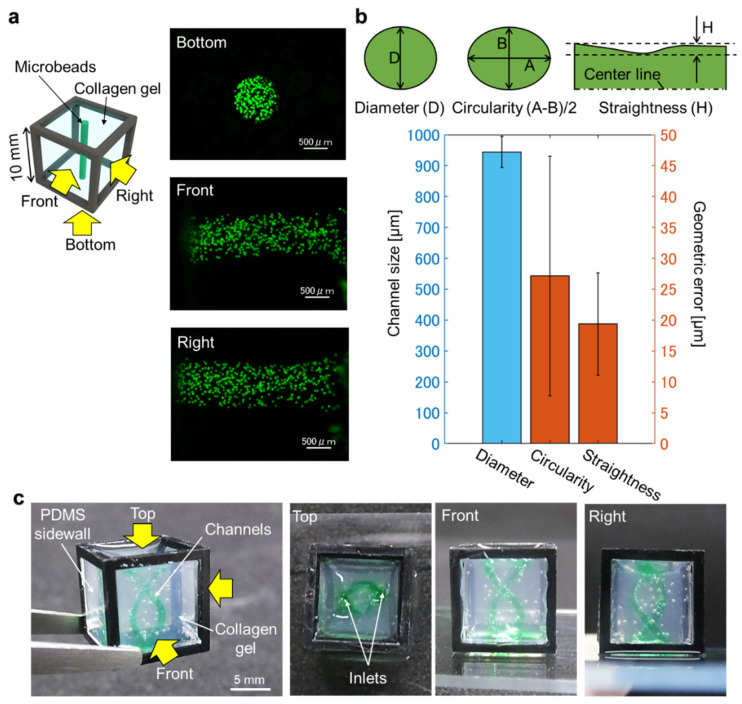
Measurement results of the collagen channel in 10 mm cube transferred from carbohydrate mold. (**a**) Schematic illustration and fluorescent images of the formed channel filled with fluorescent beads. The channel profiles were measured by scanning the filled microbeads from multi-directions. (**b**) Measurement results of the straight channel (*n* = 5). (**c**) Demonstration of helix channel in a cube filled with collagen. Water with green dye was injected into the formed channel and multi-directional views revealed the whole shape of the helix channel. Error bars denote standard deviation.

During the molding process to transfer the mold shape into the hydrogel, the printed mold is placed in the cube device followed by injection of hydrogel solution. However, as hydrogel is a soft material, it collapses easily with little mechanical stress, even under its own weight, unlike hard materials. Therefore, accuracy of the mold does not assure accuracy of the designed pattern in hydrogel. To confirm the accuracy of the transferred pattern in collagen hydrogel, a pillar-shaped mold with the same diameter as the previous experiment was used to make a shape in collagen in the cube. After the mold had been totally dissolved, fluorescent microbeads mixed with agarose gel were injected into the space where the mold had existed, and images were taken from the bottom, front, and right view of the cube to visualize and measure the shape made in the collagen gel (Figure 4a). Geometric dimensions of the transferred shape in collagen gel were measured in the same manner as in the previous experiment for mold printing accuracy. Compared with the results of the printed carbohydrate mold, the transferred pillar pattern in collagen gel had a diameter of 943.8 μm, meaning the diameter had shrunk by approximately 150 μm from the mold size (Figure 4b). Nevertheless, the standard deviation was only about 50 μm, which is relatively small in soft materials considering the fact that the whole size of the mold was 10 mm in length. The circularity and straightness of the transferred shape were 18.8 μm and 27.2 μm, respectively (Figure 4b). Given that the target size of organoids is more than 1 mm, this level of accuracy was considered good enough to enhance the multi-cellular organization.

The advantages of using carbohydrate glass molds in place of non-water-soluble molds are that complex shapes can be fabricated in the gel. To demonstrate this advantage, we developed a double-helix-shaped mold using carbohydrate glass, and transferred this shape to collagen hydrogel in a 10 mm cube device (Figure 4c). Water containing green dye was injected into the space left in the gel after dissolving the sacrificial mold to visualize the structure, and the views from three sides of the cube revealed the complex 3D structure that can be created in the gel. Without using a sacrificial mold, it would be quite difficult to generate hollow pockets with this kind of complex shape in soft materials.

### 3.4. Endothelial Cell Seeding in the Patterned Channels

**Figure 5:**
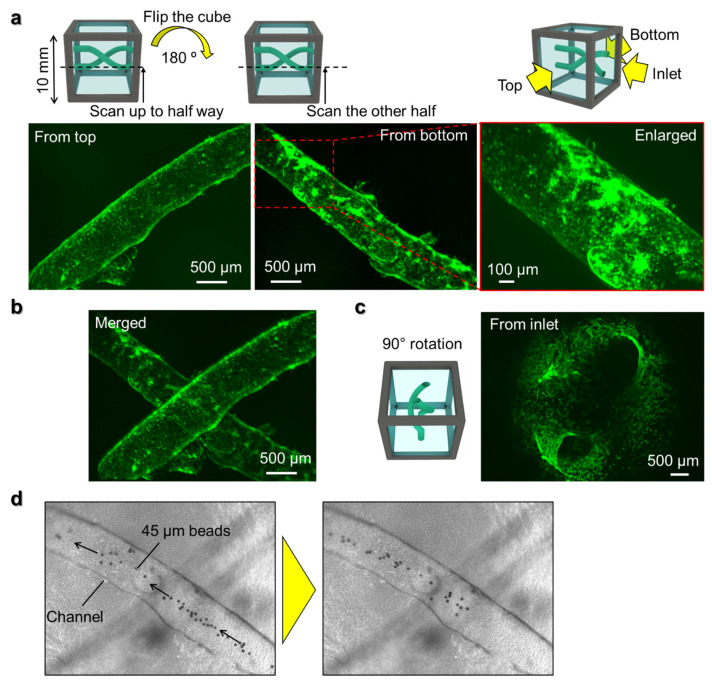
(**a**) Projection images of *z*-stack imaging from bottom half and top half with 4× magnification lens and enlarged view with 10× magnification lens. Green fluorescence shows actin filament of the HUVECs. (**b**) Projection image after superposition of the images from top and bottom. (**c**) Projection image after *z*-stack scanning from inlet by rotating the cube device at 90 °. (**d**) Microbeads of 45 μm flowing through the lumen of the channels seeded with HUVECs.

As a demonstration of how the method developed here can be applied in controlling multi-cellular formation, we seeded human umbilical vein endothelial cells (HUVEC) in the two helix-shaped channels in collagen gel in the cube, as was made above. The cells were cultured for five days, then fixed and stained with phalloidin which labels the actin filaments of the cells. Due to the long focal length when using a 10 mm cube and increasing degradation of fluorescence signal intensity the further away from the objective lens, it becomes hard to image the whole sample in the cube. To counter this problem, we first scanned halfway up the cube with 4× magnification lens to obtain *z*-stack images. Then, the cube was rotated 180° to scan the other half of the cube separately (Figure 5a). After obtaining scans from the whole cube, the images were aligned based on multiple points in the overlapped areas, then merged together (Figure 5b). The enlarged view taken with a 10× magnification lens revealed that a monolayer of endothelial cells had formed in the channel structure by connecting with neighboring cells and adhering to the remaining PLGA coating in the channels, forming a tube structure similar to blood vessel formation. By rotating the cube and merging the obtained scan images from multiple sides of the cube, the whole complex 3D structure can be visualized not only from the top and bottom (Figure 5b), but also from the side clearly showing the two holes of the inlets surrounded by HUVEC (Figure 5c). To confirm the tubular formation of the channel with HUVEC throughout the cube, 45 μm polystyrene beads were injected into the channel inlet and a video of the microbeads flowing along the HUVEC tube from the inlet to outlet of the cube was taken (Figure 5d and Supplementary Materials Video S1).

## 4. Discussion and Conclusions 

The method detailed in this paper enables the control of cell position with an unrestricted range of 3D shapes in hydrogel in a cube device by using a water-soluble mold, which we demonstrated by forming a helix-shaped vascular structure using human endothelial cells. By scanning the structure from multiple angles, large-scale high-resolution images of the whole sample could be obtained using a simple fluorescence microscope with a low magnification lens. Thus, the platform developed here has high potential to contribute to the field of organoids by offering control on organoid morphogenesis and high-resolution imaging of millimeter-sized organoids.

Current organoid culture methods mainly rely on culturing cells as a simple spheroid or cluster of cells and allowing the cells to self-organize into organoids based on specific biochemical cues from the differentiating factors in the medium. Nevertheless, the shape and geometry of the surrounding microenvironment also affect cellular migration, proliferation, and differentiation, as cells collectively react to the differential morphogen signaling gradients and mechanical stresses caused by the geometrical constraints exerted on them [19,20]. Hence, the ability to control cell shape according to the shape of the target organ may contribute to the development of more sophisticated organoids such as lung organoids with highly controlled branching formations or kidney organoids with nephrons precisely formed with the Bowman’s capsule on one end and the collecting duct on the other.

One of the remaining challenges with this method, however, is that some of the hydrophobic coating of the mold remains on the inner walls of the formed shape, and some cells may preferentially adhere to the PLGA surface rather than the hydrogel. Although PLGA is a biocompatible material and has been used in various applications such as in bone and kidney regeneration or as drug delivery carriers [21,22,23], it is still unknown if the residual PLGA may have an effect on the differentiation of stem cells such as embryonic stem cells (ESCs) and induced pluripotent stem cells (iPSCs) that are used in organoid generation, particularly as most organoid culture protocols are based on the use of soft hydrogels such as Matrigel. Reducing the concentration and coating time of the PLGA coating solution makes it easier to eliminate the PLGA residues, but doing so will compromise the accuracy and reproducibility of the shape formed in the hydrogel. Therefore, it is necessary to consider how to remove the hydrophobic coating after transferring the mold shape to the hydrogel without using harsh treatments that may cause damage to the gel or the cells. Some potential methods for removing residual PLGA are by cleaning with hydrolytic enzymes that degrade PLGA [24] after the mold has been dissolved, or by pre-mixing the degrading enzyme with the coating solution as was reported for poly(ε-caprolactone) (PCL), which is another popular hydrophobic coating polymer [25]. The optimization of such residue removal protocols for the platform developed here is currently ongoing. Another major challenge is the resolution of the mold, which depends on the diameter of the dispensing nozzle. In this research, a nozzle with a diameter of 100 µm was used, but molds with higher resolution could be attained if a nozzle with a smaller diameter was used. However, if the nozzle diameter is too small, it becomes difficult to dispense highly viscous inks such as carbohydrate glass, so the trade-off between reducing the diameter of the nozzle for high-resolution printing and printability needs to be considered.

The concept of the 3D culture platform developed in this study shows the feasibility of designing organoids with complex shapes, and the potential to analyze the morphogenetic mechanisms of organoid formation by controlling the initial position of cells. It is expected that this technology will provide new ways to develop organoids with more complex structures and functions, which can contribute to a wide range of fields such as developmental biology, drug development, and regenerative medicine.

## Data Availability

The data that support the findings of this study are available from the corresponding author upon reasonable request.

## Supplementary Materials

The following are available online at https://www.mdpi.com/article/10.3390/mi13020156/s1, Video S1: the microbeads flowing along the HUVEC tube from the inlet to outlet of the cube.
